# P11 Establishing the dose and empirical efficacy of benzylpenicillin for the treatment of severe community-acquired pneumonia

**DOI:** 10.1093/jacamr/dlaf230.018

**Published:** 2025-12-04

**Authors:** Bilal Mian, Karamjit Badyal, Kiranmai Bhatt, Aiden Plant

**Affiliations:** Dept of Microbiology, Walsall Healthcare NHS Trust, UK; Dept of Microbiology, Walsall Healthcare NHS Trust, UK; Dept of Microbiology, Black Country Pathology Services, The Royal Wolverhampton NHS Trust, UK; Dept of Microbiology, Black Country Pathology Services, The Royal Wolverhampton NHS Trust, UK

## Abstract

**Background:**

In cases of community acquired pneumonia (CAP), *Streptococcus pneumoniae* is the commonest causative pathogen and benzylpenicillin should be considered the most appropriate empirical treatment.^1^

**Objectives:**

To determine: (i) what dose of benzylpenicillin is safe in the empirical treatment of CAP, including pneumococcal pneumonia and (ii) the impact of transitioning empirical CAP treatment guidelines (for CURB-65 ≥3) from co-amoxiclav to benzylpenicillin. The dose of benzylpenicillin required to adequately treat >90% of pneumococcal isolates from blood culture was used to assess the former. Whilst the 30 and 90 day mortality was assessed 3 months pre- and post-transition to benzylpenicillin, for the latter.

**Methods:**

All cases of bloodstream infection (BSI) caused by *S. pneumoniae* were identified via the laboratory information management system. MIC determination by diffusion gradient strip testing (Etest) method were included. The MIC for all *S. pneumoniae* isolates were determined to establish the range and percentage for which benzylpenicillin at 1.2 g IV q6h would be an effective treatment. Mortality data was reviewed pre- and post-implementation of empirical CAP guideline change (November 2024) from co-amoxiclav and doxycycline to benzylpenicillin (1.2 g IV q6h) and doxycycline. The 30 and 90 day all-cause mortality for all patients with a coded first diagnosis of ‘lobar pneumonia’ and ‘pneumococcal pneumonia’ was assessed from December 2023–February 2024 (pre-intervention) and December 2024–February 2025 (post-intervention). Patients with a β-lactam allergy, or a diagnosis of seasonal influenza were excluded. Statistical analysis was performed using χ^2^.

**Results:**

There were 326 unique episodes of *S. pneumoniae* BSI in both adults and children, identified between September 2020 to November 2024. Of these, 151 isolates had evaluable MIC determination by Etest. The range of MICs was 0.004 to 1 mg/L. 98% of pneumococcal isolates had an MIC of ≤0.5 mg/L which would be amenable to treatment with benzylpenicillin at doses of 1.2 g IV q6h according to EUCAST guidance.^2^ There were 264 and 294 patients identified in the pre- and post-implementation groups respectively. Of those, 30 and 90 day mortality was 21 and 34 patients in the co-amoxiclav group, versus 27 and 38 patients in the benzylpenicillin group (difference in 30 day mortality *P*=0.62; difference in 90 day mortality *P*=0.97). Monthly antimicrobial stewardship point prevalence audits assure us that antimicrobial prescribing is >90% guideline compliant.

**Conclusions:**

Local isolates of *S. pneumoniae* identified from BSI have demonstrated that treatment with benzylpenicillin at 1.2 g IV q6h is a safe and effective in the empirical treatment of CAP. Only 2% of cases would require doses increasing to 2.4 g IV q6h (or 1.2 g IV q4h). Since introducing benzylpenicillin as first-line empirical treatment for severe CAP in replacement of co-amoxiclav, there has been no statistically significant change in all-cause mortality for patients treated in our organization. Given the threat of growing antimicrobial resistance, guideline authors should continue to critically appraise their local microbiology to ensure empirical treatment advice continues to be both as effective and narrow-spectrum as possible.^3^ This study assures our practice and the place benzylpenicillin 1.2 g IV q6h has in our current antimicrobial treatment guidelines.
